# Follicular Lymphoma Patient Relapsing with Paraneoplastic Sensory Neuronopathy (Ganglioneuropathy)

**DOI:** 10.4274/TJH-2013.0125

**Published:** 2013-09-05

**Authors:** Kadir Öztürk, Hakan Akgün, Mustafa Çakar, Yusuf Emrah Eyi, Yakup Aksoy

**Affiliations:** 1 Gülhane Military Medical Academy, Department of Gastroenterology, Ankara, Turkey

## TO THE EDITOR

Neurological symptoms can be seen in 5%-8% of lymphoma patients. The most frequent causes of neurological symptoms are herpes virus infection, neurotoxicity due to vincristine, Guillain–Barré syndrome, and the nerve involvement of the tumor [1]. Paraneoplastic polyneuropathy is a rare neurological complication of lymphoma [2]. Here we report a case of relapsed follicular lymphoma presenting with paraneoplastic sensory neuropathy (ganglioneuropathy).

**Figure 1:**
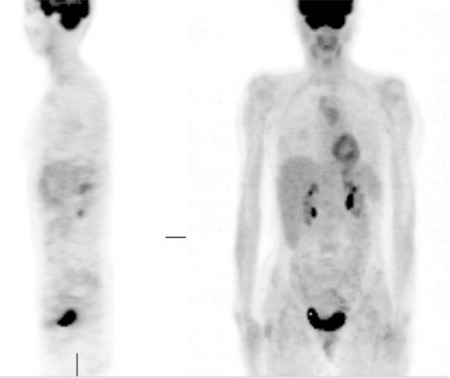
Follow-up PET reveals the remission of follicular lymphoma.

A 45-year-old woman was admitted to the hospital 2 years ago with the complaints of numbness in the hands and feet, unsteadiness, weight loss, fever, and swellings in the neck, axilla, and inguinal region. She was diagnosed with follicular lymphoma through the excisional biopsy of the cervical lymph node. She was diagnosed with stage 3B disease based on the presence of lymphadenopathies in the paratracheal, precarinal, axillary, and inguinal regions and no infiltration by bone marrow examination. The treatment started with rituximab (500 mg/day), cyclophosphamide (1125 mg/day), adriablastine (80 mg/day), vincristine (2 mg/day), and prednisolone (80 mg/day). Therapy was completed in 8 courses with 21-day intervals. After the therapy, all of the symptoms had regressed and the case was evaluated as in remission with a follow-up PET scan (Figure 1). Gradually increasing unsteadiness, numbness, and pain and burning sensation in the hands and feet developed 1 month following that PET study. She applied to the neurology service and it was found that she had normal muscle strength but mild ataxia, positive Romberg test, and prominent hypoesthesia in the distal regions of the extremities and bilateral impairment of the deep tendon reflexes and indifferent plantar responses. Informed consent was obtained.

An electromyography revealed sensorial gangliopathy. On physical examination the positioning and the vibration sensations of the patient were found to be diminished. Intravenous pulse steroid therapy (1000 mg/day) was administered for 5 days. Two days after this therapy, a severe and generalized pain developed in all of the extremities and joints. In neurological examination, distinct quadriparesis, ataxia, dysmetria, dysdiadochokinesia, hypoesthesia of the 4 extremities, and bilateral abolished deep tendon reflex with indifferent plantar responses were determined. There was neither vibration nor position sensation below the knees or elbows. Sensory-motor axonal neuropathy, which affected the motor component of the upper and lower extremities, was seen in repeated EMG. 

**Figure 2:**
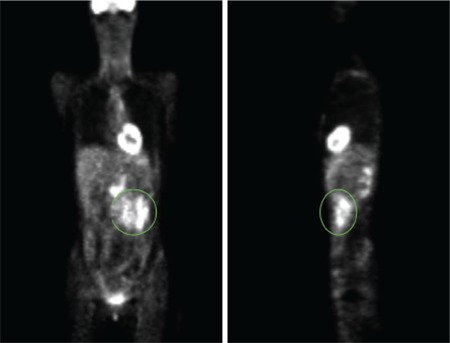
PET reveals relapse of follicular lymphoma (green circle).

Plasmapheresis therapy was performed every other day for 7 days, but the patient derived no benefit from the therapy. Abdominal ultrasonography was then performed and a tumor located in the left upper anterior region and consistent with lymph node involvement was detected. Pathological lymph nodes were seen in a the mesenteric, paraaortic, and aortocaval regions with abdominal computed tomography (CT). A tumor that was located in the left upper anterior region and consistent with lymph node involvement was also detected by PET scan (Figure 2). The levels of thyroid-stimulating hormone, vitamin B12, and folate were normal. Herpes simplex virus (HSV) antibody was negative. The levels of immunoglobulin and the lumbar puncture were inconclusive. There were no pathologic signs in the evaluation of cerebral, cervical, and thoracolumbar magnetic resonance imaging (MRI). Therefore, the patient was diagnosed with paraneoplastic polyneuropathy due to relapsed non-Hodgkin lymphoma. Dexamethasone (40 mg/day), cisplatin (50 mg/day), and cytosine arabinoside (3 g/day) were given in 3 courses. Progressive enlargement of the lymph nodes upon follow-up CT suggested tumor progression. High-dose chemotherapy (BCNU 300 mg/m2, etoposide 200 mg/m2, Ara-C 200 mg/m2, melphalan 140 mg/m2) was given and autologous stem cell transplantation (ASCT) was performed. 

Following the ASCT and high-dose chemotherapy there was an improvement in motor functions, although mild numbness of the hands and feet continued. The sensory-motor axonal polyneuropathy of the upper and lower extremities was demonstrated in follow-up EMG. 

The pathogenesis of paraneoplastic neuropathy is not clear yet, but it is attributed to autoimmune mechanisms in most cases. Both axonal and demyelinating involvement of the peripheral nerves can be seen in paraneoplastic neuropathy patients [3]. In our case, the HSV antibody was negative and there was history of vincristine usage. However, the symptoms appeared 9 months after the use of vincristine. Vincristine-induced neuropathy usually appears between weeks 2 and 18 [4]. For this reason, the neurological symptoms were thought not to occur due to use of vincristine. Guillain–Barré syndrome was eliminated as there was no albuminocytologic dissociation by the analysis of cerebrospinal fluid and there was no demyelinating disease upon EMG. As there were no pathologic signs upon cerebral, cervical, and thoracolumbar MRI evaluation, the patient was diagnosed with paraneoplastic polyneuropathy due to relapsed follicular lymphoma.

Paraneoplastic ganglioneuropathy is rare. Ganglio- neuropathy is a syndrome characterized by a symmetric/asymmetric involvement of sensorial components, diminishing or disappearance of reflexes, ataxia, glove- and stocking-type pain, and dysesthesia. These symptoms can be seen in any localization of the body, including the face. The potential of the sensory-motor nerve might be diminished while the motor transmission speed is normal or almost normal in electrophysiological studies [5]. In our case, a simultaneous axonal involvement was seen, associated with ganglioneuropathy symptoms such as clinical unsteadiness and numbness, and later on, additional motor involvement was seen. The level of vitamin B12 was normal and there was no history of alcoholism or diabetes.

In conclusion, paraneoplastic sensory neuropathy should be kept in mind in patients who present with neuropathy, particularly in the patients with malignancy. Neurotoxic drugs, infection, tumor invasion, and autoimmune disorders should be eliminated and the primary disease should be treated accordingly. 

## CONFLICT OF INTEREST STATEMENT

The authors of this paper have no conflicts of interest, including specific financial interests, relationships, and/ or affiliations relevant to the subject matter or materials included.
